# Pooled Analysis on the Effectiveness and Safety of Lipegfilgrastim in Patients With Urological Malignancies in the Real-World Setting

**DOI:** 10.3389/fonc.2021.655355

**Published:** 2021-05-28

**Authors:** Axel S. Merseburger, Götz Geiges, Jörg Klier, Martin Wiesholzer, Petra Pichler

**Affiliations:** ^1^ Department of Urology, University Hospital Schleswig-Holstein, Lübeck, Germany; ^2^ Arztpraxis für Urologie, Berlin, Germany; ^3^ Urologie Bayenthal, Köln, Germany; ^4^ Department of Internal Medicine I, University Hospital St. Poelten, Karl Landsteiner, University of Health Sciences, St. Poelten, Austria

**Keywords:** lipegfilgrastim, prostate, testicular, bladder, cancer, neutropenia, efficacy and safety

## Abstract

Lipegfilgrastim is a long-acting glycopegylated granulocyte-colony stimulating factor (G-CSF) approved for the management of chemotherapy-induced neutropenia. In general, there is little information on the use of any G-CSFs specifically in patients with urological malignancies receiving chemotherapy. This report combines information from two prospective non-interventional studies on the prophylactic use of lipegfilgrastim in urological cancer patients receiving chemotherapy in the real-world setting. Data were derived from two phase IV studies (NADIR and LEOS) with similar protocols conducted in nine European countries. Analysis included 228 patients (142 prostate, 50 testicular, 27 bladder, and 9 other urological cancers). Chemotherapy-induced febrile neutropenia risk was classified as high (43.0%), intermediate (49.1%), or low (7.5%). Lipegfilgrastim was administered as primary (*n*=180, 78.9%) or secondary (*n*=29, 12.7%) prophylaxis. The incidence of febrile neutropenia over all chemotherapy cycles (*n*=998) and first cycles (*n*=228) for which lipegfilgrastim was administered for prophylaxis was 2.6% and 1.3%, respectively. Corresponding results for Grade 3/4 neutropenia were 2.2% and 0.9%, respectively. Adverse drug reactions occurred in 24 patients (10.5%): those in more than one patient were bone pain (*n*=6, 2.6%) and pyrexia (*n*=3, 1.3%). The use of lipegfilgrastim for the prophylaxis of chemotherapy-induced neutropenia was effective and well tolerated in patients with urological malignancies in the real-world setting.

## Introduction

Chemotherapy-induced neutropenia is a common, and the most serious, hematological adverse event (AE) in cancer patients treated with myelosuppressive chemotherapies (1, 2). It frequently results in chemotherapy-dose delay or reduction, which can reduce dose intensity sufficiently to compromise optimal treatment response and survival (1, 3). Fever is frequently the first symptom of infection in cancer patients with chemotherapy-induced neutropenia and patients exhibiting febrile neutropenia usually require aggressive treatment including systemic antimicrobial treatment that necessitates hospitalization (1). Risk factors for the development of febrile neutropenia include intensive chemotherapy regimens and patient-related factors such as advanced age, advanced disease stage, previous episodes of febrile neutropenia, and comorbid conditions (4, 5).

Recombinant *N*-methionyl human granulocyte-colony stimulating factors (r-metHuG-CSFs, hereafter G-CSFs) are effective agents in cancer patients, reducing the duration and incidence of chemotherapy-induced neutropenia and febrile neutropenia by stimulating the release of neutrophils and neutrophil proliferation from bone marrow (6, 7). G-CSFs should be instituted for the prophylactic prevention of febrile neutropenia in high-risk patients (≥20% risk), and those at intermediate risk (10–20%) in the presence of other factors such as advanced age, advanced disease, previous febrile neutropenia, and no antimicrobial prophylaxis according to current treatment guidelines (4, 5, 8, 9).

G-CSFs have undergone considerable development and refinement since their initial introduction more than 25 years ago. The first agent to become available, filgrastim, has a relatively short half-life, which necessitated repeated daily subcutaneous administration for a mean of 10–11 days (10, 11) and up to 14 days per chemotherapy cycle (6, 7) to provide adequate absolute neutrophil count (ANC) recovery in clinical trials. However, a recent review of real-world observational studies showed that the mean duration of filgrastim treatment was considerably shorter at 3.7–7.5 days (12). This, combined with a delay in starting filgrastim in clinical practice, appears to contribute to reduced efficacy (13–17). The half-life of filgrastim was prolonged sufficiently by pegylation to allow the effective administration of a fixed dose of pegfilgrastim once per chemotherapy cycle (18). Single-dose pegfilgrastim was as effective as filgrastim administered daily for 10–14 days per cycle for prophylaxis of febrile neutropenia according to meta-analyses of randomized controlled trials (16, 17, 19, 20). However, observational studies suggest pegfilgrastim is more effective for the prevention of severe neutropenia and febrile neutropenia than filgrastim in a real-world setting (14, 21–23) and more convenient to administer.

Lipegfilgrastim (Lonquex^®^, Teva Pharmaceuticals Industries Ltd, Petach Tikva, Israel) is approved by the European Medicines Agency for the management of chemotherapy-induced neutropenia (24). It is a glycopegylated G-CSF with a prolonged half-life compared with the same dose of pegfilgrastim and produces a more sustained ANC increase (25–27). Lipegfilgrastim was at least as effective and as well tolerated compared to the same dose of pegfilgrastim in a phase III clinical trial among breast cancer patients receiving doxorubicin/docetaxel (28); subsequent analysis of secondary endpoints revealed a favorable response with lipegfilgrastim for some endpoints (e.g. time to ANC recovery, incidence of severe neutropenia, depth of ANC nadir) (26, 29). Glycopegylation as applied for lipegfilgrastim is the most specific pegylation method available to date, selectively adding one molecule of methoxyPEG (mPEG) to a sugar-activated threonine (threonine^134^) in filgrastim. In addition to this defined glycopegylation pattern, the position of the methoxypegylation site is also worthy of mention and could be the basis for hypothesis generation, as one particular aspect of the PEG modification in lipegfilgrastim is shielding the protein from enzymatic degradation and protection from recognition by the immune system. A centrally positioned mPEG-chain, as in lipegfilgrastim, might be favorable over an *N*-terminal-positioned PEG-chain as in pegfilgrastim (26).

There are no published reports on the effectiveness or safety of lipegfilgrastim specifically in patients with urological malignancies such as prostate, testicular, kidney, or bladder cancer. Patients with testicular cancer are usually young and are treated with highly myelosuppressive chemotherapy regimens where the goal is to cure the disease and, consequently, it is highly important to maintain dose intensity. Many cisplatin-based chemotherapy regimens for testicular cancer have a high risk, e.g., VelP (vinblastine, ifosfamide, cisplatin), VIP (etoposide, ifosfamide, cisplatin), BEP (bleomycin, etoposide, cisplatin), TIP (paclitaxel, ifosfamide, cisplatin) (30–33), or intermediate risk for febrile neutropenia, e.g., etoposide/cisplatin (34). Dose-dense MVAC (methotrexate, vinblastine, cyclophosphamide, cisplatin) in bladder cancer (35) presents a high risk for febrile neutropenia. Cabazitaxel plus prednisone presents an intermediate risk for febrile neutropenia in castration-resistant prostate cancer (36). Many of the patients with an intermediate risk of developing febrile neutropenia have additional factors that increase their risk, e.g. advanced age and comorbidities, making them candidates for G-CSF treatment. Here, we report pooled data from two non-interventional studies of lipegfilgrastim conducted in urological cancer patients with chemotherapy-induced neutropenia.

## Methods

Data were derived from two (NADIR and LEOS) prospective, multicenter, phase IV, observational cohort studies with similar protocols, which were conducted from 8 January 2014 to 16 January 2017 in nine European countries (Austria, Belgium, Czech Republic, Germany, Italy, Poland, Slovakia, Spain, and The Netherlands). Neither study had any influence on an individual’s course of treatment. The study protocols were approved by ethical review boards at all their respective study centers and written informed consent was obtained from patients prior to inclusion. The studies followed the principles of the Declaration of Helsinki and guidelines of the International Conference on Harmonization of Good Clinical Practice. Full details of the methods and results of NADIR have been published for all 2,489 evaluable patients with various cancers (37). Interim results of the LEOS study have been reported for 621 patients with various cancers (38).

### Patients and Treatment

Patients were male or female adults (≥18 years) with solid urological tumors receiving cytotoxic chemotherapy in whom their treating physician had decided to institute lipegfilgrastim for primary or secondary prophylaxis of neutropenia. Pregnant or lactating women were excluded.

The treating physician determined the cancer therapy appropriate for each patient, which followed the standard clinical practice for the relevant country. Lipegfilgrastim 6 mg was administered subcutaneously approximately 24 h after cytotoxic chemotherapy and continued for up to six cycles according to standard clinical practice and within approved marketing authorization (37).

### Assessments

The following baseline demographic and clinical characteristics were recorded during screening: age, gender, height, weight, ethnicity, Eastern Cooperative Oncology Group (ECOG) performance status score, primary disease/tumor history, nutritional deficiency, details of previous and current chemotherapy, history of febrile neutropenia, concurrent diseases and medications, and blood counts. Baseline individual patient-related risk factors for febrile neutropenia (4) and intended use of lipegfilgrastim (primary or secondary prophylaxis) were determined. Patients were followed from initiation of lipegfilgrastim until 6–8 weeks after the last dose of lipegfilgrastim. Efficacy endpoints included incidence of severe (Grade 3/4) neutropenia, febrile neutropenia, use of antimicrobial agents, hospitalization, and chemotherapy dose modifications.

### Safety

AEs and adverse drug reactions (ADRs) potentially related to lipegfilgrastim (yes/no) were coded by Medical Dictionary for Regulatory Affairs v20.0 and graded by National Cancer Institute Common Terminology Criteria for Adverse Events v4.03. Serious AEs and serious ADRs were reported in an expedited manner and coded/graded in the same manner. Highly prevalent chemotherapy-derived AEs (e.g., nausea, vomiting) were excluded from being recorded in one study (LEOS).

### Statistics

Data are reported using descriptive statistics with continuous variables as mean ± standard deviation (SD) and median (range), and different categories of discrete variables as frequencies (%). Statistical analyses were performed using IBM^®^ SPSS Statistics (v21.0 or higher) and StatXact^®^ (v6.0), with missing values not being replaced or extrapolated.

## Results

### Demographics and Clinical Characteristics

The study included a total of 228 patients who had received ≥2 cycles of chemotherapy with at least one dose of lipegfilgrastim, which constituted the safety and efficacy populations. The demographic and clinical characteristics of the entire study population (*N*=228) as well as the three major subpopulations with prostate (*n*=142), testicular (*n*=50), and bladder (*n*=27) cancer are summarized in **Table 1**. Age and tumor stage were lower in the subpopulation with testicular cancer.

**Table 1:** Demographics and clinical characteristics.

	All patients (*N*=228)	Prostate cancer (*n*=142)	Testicular cancer (*n*=50)	Bladder cancer (*n*=27)
Mean ± SD age, years (range)	63.7 ± 15.6 (21–89)	70.9 ± 7.7 (48–89)	39.1 ± 10.7 (21–61)	70.7 ± 9.4 (46–87)
Sex, *n* (%)				
Male	220 (96.5)	142 (100)	50 (100)	19 (70.4)
Female	8 (3.5)	0	0	8 (29.6)
ECOG performance status score				
0	117 (51.3)	71 (50.0)	30 (60.0)	13 (48.1)
1	88 (38.6)	53 (37.3)	18 (36.0)	12 (44.4)
2	11 (4.8)	9 (6.3)	1 (2.0)	1 (3.7)
≥3	1 (0.4)	1 (0.7)	0	0
Data missing	11 (4.8)	8 (5.6)	1 (2.0)	1 (3.7)
Mean ± SD time from diagnosis to inclusion, year (range)	4.11 ± 5.16 (0.01–26.51)	5.86 ± 5.22 (0.02–24.55)	1.14 ± 4.29 (21–61)	1.63 ± 2.41 (0.02–10.74)
Primary tumor, *n* (%)				
Prostate	142 (62.3)	142 (100)	–	–
Testicular	50 (21.9)	–	50 (100)	–
Bladder	27 (11.8)	–	–	27 (100)
Other^a^	9 (4.4)	–	–	–
Previous cancer treatment				
Chemotherapy	167 (73.2)	104 (73.2)	50 (100)	23 (85.2)
Radiotherapy	69 (30.3)	64 (45.1)	0	4 (14.8)
Hormonal	38 (16.7)	38 (26.8)	0	0
Other^b^	1 (0.4)	1 (0.7)	0	0
Tumor stage, *n* (%)^c^				
I	8 (5.4)	0	6 (16.7)	1 (5.0)
II	37 (25.0)	16 (18.2)	17 (47.2)	3 (15.0)
III	30 (20.3)	14 (15.9)	12 (33.3)	3 (15.0)
IV	73 (49.3)	58 (65.9)	1 (2.8)	13 (65.0)
Data missing	80	54	14	7
Metastatic stage, *n* (%)				
M_0_	177 (77.6)	109 (76.7)	37 (74.0)	22 (81.4)
M_1_	37 (16.2)	27 (19.0)	7 (14.0)	3 (11.1)
M_x_	9 (3.9)	2 (1.4)	5 (10.0)	2 (7.4)
Data missing	5 (2.2)	4 (2.8)	1 (2.0)	0

a Includes urothelial (n=4), kidney (n=2), ureter (n=2), and penis (n=1).

b Includes immunotherapy, targeted therapy, or biologic therapy.

c Missing data not included in percentage calculation.

Percentages may not total 100% exactly due to rounding.

ECOG, Eastern Cooperative Oncology Group; SD, standard deviation.

Overall, ~60% of the patients had ≥1 comorbidity, with 25.6% having 1, 20.7% having 2, 10.1% having 3, and 2.6% having ≥4 concurrent comorbidities (**Table 2**). The mean number of comorbidities per patient was 1.09 ± 1.15, overall, but was considerably lower at 0.54 ± 0.73 in the subgroup with testicular cancer.

**Table 2:** Comorbidities.

	All patients (*N*=227)	Prostate cancer (*n*=141)	Testicular cancer (*n*=50)	Bladder cancer (*n*=27)
No. of patients with comorbidities, *n* (%)	134 (59.0)	89 (63.1)	20 (40.0)	17 (63.0)
Mean ± SD no. of comorbidities per patient, *n* (%)	1.09 ± 1.15 (0–6)	1.08 ± 1.15 (0–4)	0.54 ± 0.73 (0–2)	1.33 ± 1.44 (0–6)
No. of comorbidities, *n* (%)				
0	93 (41.0)	52 (36.9)	30 (60.0)	10 (37.0)
1	58 (25.6)	38 (27.0)	13 (26.0)	6 (22.2)
2	47 (20.7)	30 (21.3)	7 (14.0)	6 (22.2)
3	23 (10.1)	16 (11.3)	0	4 (14.8)
≥4	6 (2.6)	5 (3.5)	0	1 (3.7)
Comorbidity by SOC, *n* (%)				
Cardiovascular	91 (40.1)	62 (44.0)	8 (16.0)	15 (55.6)
Endocrine	36 (15.9)	24 (17.0)	3 (6.0)	5 (18.5)
Musculoskeletal	10 (4.4)	7 (5.0)	1 (2.0)	1 (3.7)
CNS	8 (3.5)	6 (4.3)	1 (2.0)	1 (3.7)
Digestive	8 (3.5)	2 (1.4)	2 (4.0)	3 (11.1)
Genitourinary	7 (3.1)	3 (2.1)	2 (4.0)	2 (7.4)
Peripheral nervous system	4 (1.8)	3 (2.1)	1 (2.0)	0
Respiratory	4 (1.8)	3 (2.1)	1 (2.0)	0
Other	79 (35.0)	56 (39.7)	8 (16.0)	9 (33.3)

Percentages may not total 100% exactly due to rounding.

CNS, central nervous system; SD, standard deviation; SOC, system organ class.

Details of neutropenic risk, chemotherapies, and prophylaxis type are summarized in **Table 3** (35, 36, 39–50). Overall, the mean number of patient-related risk factors for febrile neutropenia for the entire population was 1.36 ± 0.96; age >65 years (*n*=136, 59.6%), prior febrile neutropenia (*n*=116, 50.9%), and hemoglobin <12 g/dL (*n*=42, 18.4%) accounted for nearly all of the risk factors for febrile neutropenia. Chemotherapy-induced febrile neutropenia risk was intermediate (49.1%) or high (43.0%) in nearly all patients, with 7.5% having low risk. The chemotherapy setting was usually palliative (58.3%) or metastatic (18.4%), which would appear to be related to the high prevalence of patients with prostate cancer in the total cohort. The subpopulation with testicular cancer had a lower mean number of risk factors for febrile neutropenia (0.40 ± 0.61), more frequently had a high risk of chemotherapy febrile neutropenia (64.0%), and more frequently had a curative (26.0%), adjuvant (20.0%), or adjuvant/induction setting (24.0%) relative to palliative (22.0%) or metastatic settings (8.0%). Lipegfilgrastim was administered as primary prophylaxis in 180 patients (78.9%) and secondary prophylaxis in 29 (12.7%), with missing data in 19 (8.3%): there was a similar distribution across the three cancer subpopulations.

**Table 3:** Neutropenic risk, chemotherapies, and prophylaxis type.

	All patients(*N*=228)	Prostate cancer(*n*=142)	Testicular cancer(*n*=50)	Bladder cancer(*n*=27)
Mean ± SD no. of patient-associated risk factors for febrile neutropenia	1.36 ± 0.96	1.67 ± 0.80	0.40 ± 0.61	1.63 ± 0.97
Patient-associated risk factors for febrile neutropenia, *n* (%)				
Age >65 years	136 (59.6)	108 (76.1)	0	21 (77.8)
Prior febrile neutropenia	116 (50.9)	88 (62.0)	14 (28.0)	12 (44.4)
Hemoglobin <12 g/dL	42 (18.4)	33 (23.2)	5 (10.0)	4 (14.8)
Female sex	7 (3.1)	–	–	7 (25.9)
Poor nutritional status	5 (2.2)	4 (2.8)	1 (2.0)	0
Poor performance status	4 (1.8)	4 (2.8)	0	0
Planned chemotherapy, *n* (%) [febrile neutropenia risk with references]^a^				
Cabazitaxel [8% (36)]	73 (32.0)	73 (51.4)	0	0
Docetaxel [6% (39)]	51 (22.4)	50 (35.2)	0	1 (3.7)
Bleomycin/etoposide/cisplatin [19% (40)]	44 (19.3)	0	44 (88.0)	0
Cisplatin/gemcitabine [2% (41,42)]	10 (4.4)	1 (0.7)	0	7 (25.9)
Carboplatin/gemcitabine [NR (43)]	5 (2.2)	0	0	3 (11.1)
Docetaxel/prednisolone [3% (44)]	5 (2.2)	5 (3.5)	0	0
Vinflunine [<10% (45)]	5 (2.2)	0	0	3 (11.1)
Carboplatin/etoposide [NR]	4 (1.8)	1 (0.7)	0	2
Cabazitaxel/prednisone [2.4% (46); 10–20% (36)]	3 (1.3)	3 (2.1)	0	0
Carboplatin/paclitaxel [NR (47)]	3 (1.3)	1 (0.7)	0	1
Docetaxel/prednisone [3% (44)]	3 (1.3)	3 (2.1)	0	0
Gemcitabine/paclitaxel [19% (48)]	3 (1.3)	1 (0.7)	0	2 (7.4)
Gemcitabine [3% (49)]	3 (1.3)	0	0	3 (11.1)
Bleomycin [NR]	2 (0.9)	0	2 (4.0)	0
Carboplatin/gemcitabine/dexamethasone [NR]	2 (0.9)	0	0	1 (3.7)
Methotrexate/vinblastine/doxorubicin/cisplatin [14% (35,41,42,50)]	2 (0.9)	0	0	2 (7.4)
Other^b^	11 (4.8)	4 (2.8)	4 (8.0)	3 (70.3)
Chemotherapy setting, *n* (%)				
Palliative	133 (58.3)	101 (71.1)	11 (22.0)	16 (59.3)
Metastatic	42 (18.4)	35 (24.6)	4 (8.0)	3 (11.1)
Adjuvant	15 (6.6)	0	10 (20.0)	2 (7.4)
Adjuvant/induction	14 (6.1)	1 (0.7)	12 (24.0)	1 (3.7)
Curative	14 (6.1)	0	13 (26.0)	1 (3.7)
Other^c^	7 (3.1)	3 (2.1)	0	4 (14.8)
Data missing	3 (1.3)	2 (1.4)	0	0
Chemotherapy febrile neutropenia risk, *n* (%)				
Low (<10%)	17 (7.5)	10 (7.0)	3 (6.0)	3 (11.1)
Intermediate (10–20%)	112 (49.1)	77 (54.2)	15 (30.0)	15 (55.6)
High (>20%)	98 (43.0)	54 (38.0)	32 (64.0)	9 (33.3)
Data missing	1 (0.4)	1 (0.7)	0	0
Prophylaxis type for lipegfilgrastim use, *n* (%)				
Primary	180 (78.9)	120 (84.5)	42 (84.0)	15 (55.6)
Secondary	29 (12.7)	16 (11.3)	5 (10.0)	5 (18.5)
Data missing	19 (8.3)	6 (4.2)	3 (6.0)	7 (25.9)

a Includes single-agent chemotherapy regimens in which G-CSF use is not common, as well as chemotherapy regimens in which there is a paucity of data to support long-acting G-CSF use during the time in which chemotherapy agents may affect the bone marrow.

b Includes bleomycin/etoposide/cisplatin to etoposide/cisplatin, cabazitaxel/prednisolone, cabazitaxel/prednisone/methotrexate/dexamethasone, carboplatin, cisplatin/etoposide/dexamethasone, cisplatin/paclitaxel, docetaxel/cabazitaxel, docetaxel/cisplatin/5-fluoruracil, mitoxantrone, and vinblastine/ifosfamide/cisplatin (each n=1).

c Includes neoadjuvant (n=3), neoadjuvant/consolidation (n=2), and maintenance (n=1).

Percentages may not total 100% exactly due to rounding.

NR, not reported; SD, standard deviation.

### Effectiveness

The incidence of febrile neutropenia over all cycles (*n*=998) and first cycles (*n*=228) that lipegfilgrastim was administered for prophylaxis was 2.6% and 1.3%, respectively (**Table 4**). The incidence of febrile neutropenia over all cycles and first cycles, respectively, was lower among those who received primary prophylaxis (1.7% and 0.6%) compared to those who received secondary prophylaxis (6.9% and 3.4%). The incidence of Grade 3/4 neutropenia over all cycles and first cycles of lipegfilgrastim was 2.2% and 1.3%, respectively, with lower incidences during primary prophylaxis (2.2% and 1.1%, respectively) compared to secondary prophylaxis (3.4% and 3.4%, respectively).

**Table 4:** Incidence of febrile neutropenia and neutropenia over all cycles and in the first cycle according to intention of lipegfilgrastim treatment.

	All patients (*N*=228)	Primary prophylaxis (*n*=180)	Secondary prophylaxis (*n*=29)	Data missing (*n*=19)
All cycles, *n*	998	817	120	61
Febrile neutropenia, no. of patients (%)	6 (2.6)	3 (1.7)	2 (6.9)	1 (5.3)
Grade 3/4 neutropenia, no. of patients (%)	5 (2.2)	4 (2.2)	1 (3.4)	0
First cycles, *n*	228	180	29	19
Febrile neutropenia, no. of patients (%)	3 (1.3)	1 (0.6)	1 (3.4)	1 (5.3)
Grade 3/4 neutropenia, no. of patients (%)	2 (0.9)	2 (1.1)	0	0

Anti-infective drug use was evaluable over 834 of 998 cycles (83.8%) during which lipegfilgrastim was administered as prophylaxis. Anti-infective agents were used in 15 patients (6.6%) overall, including 11 (6.1%) during primary prophylaxis and four (13.8%) during secondary prophylaxis. The route of anti-infective drug administration was oral (*n*=11) or intravenous (*n*=4). The reasons for anti-infective drug use were prophylaxis (*n*=7), cystitis (*n*=1), fever without neutropenia (*n*=1), hematuria (*n*=1), pharyngitis (*n*=1), pharyngo-laryngitis (*n*=1), and unknown (*n*=3). No antimycotic agents were used.

### Safety

Fifty ADRs (12.5%) occurred in 24 patients (10.5%). The most frequent ADRs (occurring in >1% of patients) were bone pain (*n*=6, 2.6%) and pyrexia (*n*=3, 1.3%). The full list of ADRs is reported in **Table 5**. Lipegfilgrastim was discontinued because of an ADR in one patient (0.4%). Fourteen serious ADRs (6.1%) were recorded in nine patients (3.9%). The most frequent serious ADRs (occurring in >1 patient) were fever and hematuria (each *n*=2, 0.9%). Overall, 12 serious AEs were categorized as deaths occurring in nine patients (3.95%), none of which were considered related to lipegfilgrastim. The events leading to death in the nine patients were reported as prostate cancer with metastasis and general physical health deterioration, sepsis, general physical health deterioration and neoplasm, general physical health deterioration and anemia, renal failure, *Clostridium difficile* colitis, neoplasm recurrence, pneumonia, and death.

**Table 5:** Adverse reactions coded in System Organ Classes and Preferred Terms.

SOC Term	PT Term	Number of AE	% of AE	Number of patients (N=228)	% of patients (N=228)
Blood and lymphatic system disorders	Leukocytosis	1	2.0	1	0.4
	Neutropenia	2	4.0	2	0.9
Eye disorders	Vitreous floaters	1	2.0	1	0.4
Gastrointestinal disorders	Abdominal pain upper	3	6.0	1	0.4
	Constipation	1	2.0	1	0.4
	Enteritis	1	2.0	1	0.4
	Nausea	3	6.0	1	0.4
	Oesophagitis	1	2.0	1	0.4
General disorders and administration site conditions	Asthenia	1	2.0	1	0.4
	Fatigue	1	2.0	1	0.4
	Pyrexia	3	6.0	3	1.3
Infections and infestations	*Campylobacter* gastroenteritis	1	2.0	1	0.4
	Cystitis	1	2.0	1	0.4
	Infection	1	2.0	1	0.4
	Pharyngitis	1	2.0	1	0.4
	Pneumonia	1	2.0	1	0.4
	Rhinitis	1	2.0	1	0.4
	Sepsis	1	2.0	1	0.4
	Urinary tract infection	1	2.0	1	0.4
Injury, poisoning and procedural complications	Stenosis of vesicourethral anastomosis	1	2.0	1	0.4
Metabolism and nutrition disorders	Hypocalcaemia	1	2.0	1	0.4
	Hypomagnesaemia	1	2.0	1	0.4
Musculoskeletal and connective tissue disorders	Arthralgia	4	8.0	2	0.9
	Bone pain	6	12.0	6	2.6
	Growing pains	1	2.0	1	0.4
	Myalgia	2	4.0	2	0.9
Nervous system disorders	Headache	1	2.0	1	0.4
Psychiatric disorders	Insomnia	1	2.0	1	0.4
Renal and urinary disorders	Chronic kidney disease	1	2.0	1	0.4
	Haematuria	2	4.0	1	0.4
Respiratory, thoracic and mediastinal disorders	Pneumonitis	1	2.0	1	0.4
Skin and subcutaneous tissue disorders	Alopecia	1	2.0	1	0.4
	Skin dystrophy	1	2.0	1	0.4
Vascular disorders	Deep vein thrombosis	1	2.0	1	0.4
	Total	50	100.0	24	10.5

## Discussion

The incidence of febrile neutropenia and Grade 3/4 neutropenia was 2.6% and 2.2%, respectively, over all cycles (*n*=998) of chemotherapy when lipegfilgrastim was administered as prophylaxis for neutropenia in a cohort of 228 patients with various urological malignancies (predominantly prostate, testicular, and bladder cancers). The corresponding incidence of febrile neutropenia and Grade 3/4 neutropenia was 1.3% and 0.9%, respectively, when analysis was restricted to the first cycle of chemotherapy. The incidence of febrile neutropenia was lower at 1.7% over all cycles, and even lower at 0.6% over the first cycle, when analysis was restricted to those patients who received lipegfilgrastim as primary prophylaxis. Final results from the full NADIR study using lipegfilgrastim in ~2500 patients with a range of cancers (predominantly breast cancer, lung cancer, and non-Hodgkin lymphoma) showed an almost identical incidence of febrile neutropenia (2.7%), while the incidence of Grade 3/4 neutropenia was considerably higher at 26.8% (37).

Lipegfilgrastim and pegfilgrastim (each 6 mg per cycle) have been compared directly in a prospective, randomized, controlled trial in 188 patients with breast cancer receiving up to 4 cycles of high-risk chemotherapy (28). None of the lipegfilgrastim recipients experienced febrile neutropenia during cycle 1 compared with 3.2% of pegfilgrastim recipients and the incidence of severe (Grade 4) neutropenia was 43.6% and 51.1% (*p*=0.3409), respectively, during cycle 1. Lipegfilgrastim 6 mg and placebo have also been compared in a prospective, randomized, controlled trial in ~375 patients with non-small cell lung cancer receiving up to 4 cycles of high-risk chemotherapy (51). The incidence of febrile neutropenia was 2.4% and 5.6% with lipegfilgrastim and placebo, respectively, during cycle 1.

The incidence of febrile neutropenia has been reported in a number of observational studies of pegfilgrastim: 3% (52), 4.4% (51, 52), 5.7% (53), and 5.6–6.3% (54). These observational studies rarely included any patients with urological malignancies and none reported results specifically in this subpopulation. Pegfilgrastim and filgrastim have been compared in many observational studies but such studies did not include patients with urological malignancies and several did not report the incidence of febrile neutropenia or severe neutropenia. Morrison and colleagues (21) reported a lower incidence of febrile neutropenia comparing pegfilgrastim and filgrastim in 2,863 US patients (4.7% *vs* 6.5%, *p*=0.044), and Almenar Cubells and colleagues (14) reported respective incidences for febrile neutropenia (6.7% *vs* 13.3%) and Grade 3/4 neutropenia (28.3% *vs* 49.3%) among 391 Spanish patients. A recent review of real-world comparative effectiveness studies found that the risk of febrile neutropenia was generally lower for prophylaxis with pegfilgrastim compared with that with short-acting G-CSFs (12).

It should be noted that 43.0% of the study population of patients with urological malignancies had a high risk (>20%) of febrile neutropenia from the chemotherapy compared to 49.1% with an intermediate risk (10–20%) and 7.5% with a low risk (<10%). Almost 80% of patients received lipegfilgrastim as primary prophylaxis. This would generally appear to be in line with current treatment guidelines which recommend primary G-CSF prophylaxis should be given to patients at high risk and to those at intermediate risk in the presence of other risk factors such as advanced age, advanced disease stage, prior febrile neutropenia, and/or concurrent comorbid conditions (4, 5, 8, 9). Most patients had additional risk factors [including age >65 years (59.6%), prior febrile neutropenia (50.9%), and hemoglobin >12 g/dL (18.4%)], at least one comorbidity (59.0%), and stage III/IV disease (69.6%), which would account for the use of lipegfilgrastim in those patients who received intermediate- or low-risk chemotherapy regimens.

With respect to safety, any grade ADRs occurred in 15.4% of patients, with bone pain (2.6%) and pyrexia (1.3%) occurring in >1% of patients. The ADR profile was similar to that previously reported for lipegfilgrastim in other cancers and compared to other long- and short-acting G-CSFs (28, 55).

The study was limited by the relatively low numbers of patients evaluated, although this is the first study to our knowledge that has specifically examined the efficacy and safety of lipegfilgrastim in patients with urological malignancies. The number of patients receiving lipegfilgrastim as secondary prophylaxis in the study was too low to allow any meaningful or reliable conclusions with respect to effectiveness. The study was, furthermore, limited by the relatively large proportion of patients with missing data. Another limitation of the investigation is the use of reduced Cabazitaxel dose to 20 mg/kg, which is mainly carried out in the USA. However, in real-life clinical scenarios Cabazitaxel is also often dose reduced so that there are fewer side effects whilst maintaining similar efficacy, as shown in recent publications (56).

## Conclusion

In conclusion, lipegfilgrastim was effective and well tolerated for the prophylaxis of chemotherapy-induced neutropenia in patients with urological malignancies in the real-world setting. The use of lipegfilgrastim in these patients generally appeared to comply with current treatment recommendations and guidelines.

## Data Availability Statement

The original contributions presented in the study are included in the article/supplementary material. Further inquiries can be directed to the corresponding author.

## Author Contributions

All authors contributed to the article and approved the submitted version.

## Funding

This work was sponsored by Teva Pharmaceuticals Europe B.V. (Amsterdam, The Netherlands).

## Conflict of Interest

GG was employed by Arztpraxis für Urologie. AM declares receiving lecture or speaker fees, or honoraria from AstraZeneca, Bristol-Myers Squibb, Eisai, Ipsen, MSD, Merck Serono, Janssen, Takeda, Teva, Astellas, Novartis, Pfizer, and Roche; has acted as a consultant for AstraZeneca, Astellas, Bristol-Myers Squibb, Ipsen, Janssen, EUSAPharm, MSD, Merck Serono, Novartis, Takeda, Teva, Pfizer, and Roche; and has conducted research or clinical trials for AstraZeneca, Astellas, Bristol-Myers Squibb, Ipsen, Janssen, EUSAPharm, MSD, Merck Serono, Novartis, Takeda, Teva, Pfizer, and Roche.

The authors declare that this study received funding from Teva Pharmaceuticals Europe B.V. (Amsterdam, The Netherlands). The funder had the following involvement in the study: study design, conduct, and data analysis.

The remaining authors declare that the research was conducted in the absence of any commercial or financial relationships that could be construed as a potential conflict of interest.
